# RELATIONSHIPS BETWEEN PAIN AND OPTIMISM AMONG COMMUNITY DWELLING OLDER ADULTS

**DOI:** 10.1093/geroni/igad104.2933

**Published:** 2023-12-21

**Authors:** Lucia Rivera, Isabel Mancilla, Jaclyn Bergstrom, Anthony Molina

**Affiliations:** University of California, San Diego, San Diego, California, United States; University of California San Diego, San Diego, California, United States; University of California, San Diego, San Diego, California, United States; University of California, San Diego, San Diego, California, United States

## Abstract

Measures of life outlook in older adults have been investigated in connection to pain, as both pain management and outlook are important factors of successful aging. Positive outlook helps promote healthy aging while depression is associated with higher pain levels and interference, which negatively contribute to the aging process. Thus, we hypothesized that higher pain levels are associated with lower optimism among community-dwelling older adults. We utilized data from the UC San Diego Successful Aging Evaluation (SAGE), a prospective longitudinal cohort study initiated in 2010, to evaluate the relationship between pain and optimism in 378 community-dwelling adults aged ≥50 years. We used the revised Life Orientation Test (LOT-R) to measure optimism and three pain subscales, PROMIS Pain Interference, PROMIS Pain Intensity, and MOS 36-Item Short-Form Health Survey (SF-36), as pain measures. Regression analyses reveal negative relationships between pain and optimism for all three pain scales, with regression coefficients of -0.277 (p< 0.0001), -0.246 (p< 0.0001), and 0.269 (p< 0.0001) respectively. The correlations were strongest among men, Caucasians, and college graduates. The correlations remained significant when adjusting for gender, age, completion of college, marriage status, and household income, but not for race/ethnicity. This study was limited by the underrepresentation of non-Caucasian participants (N=87) and by the self-reported nature of the SAGE data. These findings indicate value in considering physical and psychological elements together when encouraging healthy aging. Future research can investigate whether interventions that increase optimism can decrease pain in older adults or whether pain management interventions can increase optimism in this population.

